# Supplementary figures and images for: Effect of Black Tea Infusion on Physicochemical Properties, Antioxidant Capacity and Microstructure of Acidified Dairy Gel during Cold Storage
Source: Foods. 2020 Jun 25;9(6):831. doi: 10.3390/foods9060831 (PMC7353655; doi:10.3390/foods9060831)

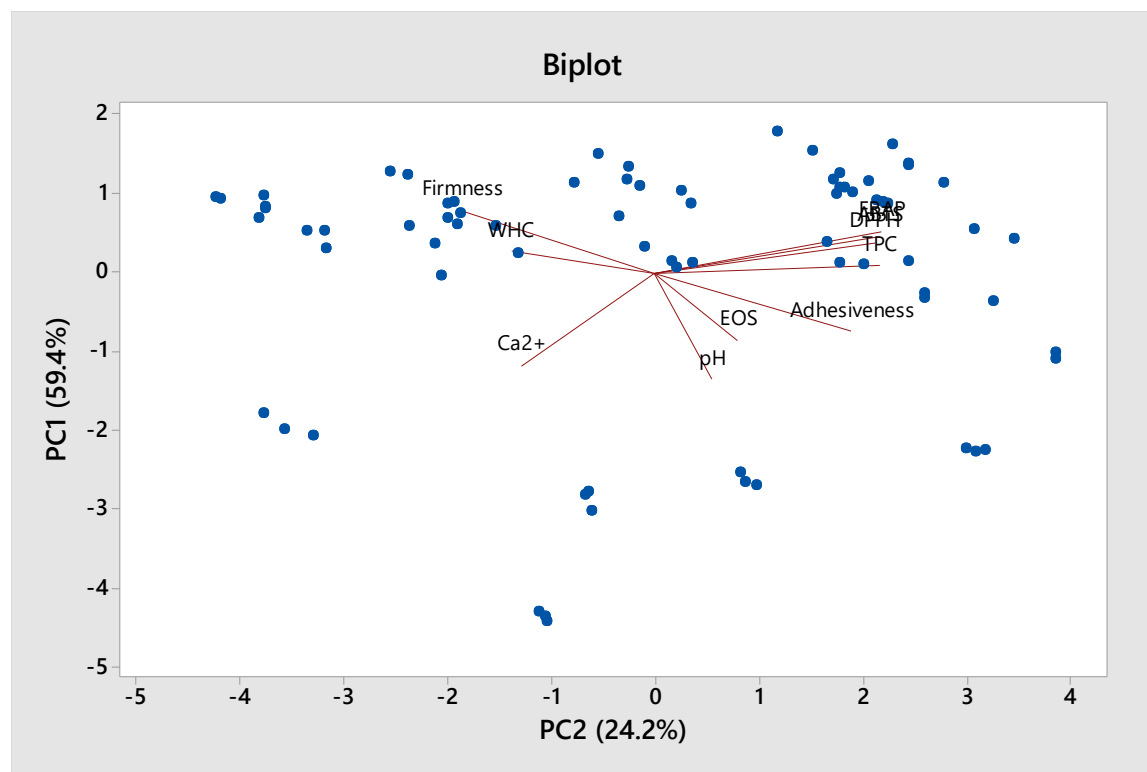

**Supplement Figure S1.** Biplot of the PCA

Supplement: Supplementary file 1 [file foods-09-00831-s001.pdf]
